# Knowledge, Perception, and Practices of Wildlife Conservation and Biodiversity Management in Bangladesh

**DOI:** 10.3390/ani15030296

**Published:** 2025-01-21

**Authors:** Raf Ana Rabbi Shawon, Md. Matiur Rahman, Samuel Opoku Dandi, Ben Agbayiza, Md Mehedi Iqbal, Michael Essien Sakyi, Junji Moribe

**Affiliations:** 1 Laboratory of Wildlife Resources, Gifu University, Gifu 501-1193, Japan; rafana.shawon@gmail.com; 2 Laboratory of Food and Environmental Hygiene, Gifu University, Gifu 501-1193, Japan; matiur.vetmed@gmail.com (M.M.R.); kessien98@gmail.com (M.E.S.); 3 Department of Medicine, Sylhet Agricultural University, Sylhet 3100, Bangladesh; 4 Department of Aquaculture and Fisheries Sciences, Faculty of Biosciences, University for Development Studies, Tamale P.O. Box TL 1350, Ghana; dandisam555@gmail.com; 5 Department of Biotechnology and Molecular Biology, Faculty of Biosciences, University for Development Studies, Tamale P.O. Box TL 1350, Ghana; ben.agbayiza@uds.edu.gh; 6 Institute for East China Sea Research, Nagasaki University, Nagasaki 851-2213, Japan; mehedi.imsf@gmail.com

**Keywords:** Bangladesh, sociodemographic, students, local communities, safeguarding, wildlife conservation

## Abstract

Human activities significantly influence the changes in wildlife habitats and biodiversity, highlighting the necessity to enhance public knowledge, perceptions, and practices (KPP) to mitigate their consequences. Previous research suggests that many people in Bangladesh do not consider wildlife and biodiversity conservation a crucial issue. However, enhancing their KPP could be essential in developing effective and sustainable conservation efforts in Bangladesh. So, the current study aimed to evaluate the public’s KPP of wildlife conservation and biodiversity management in Bangladesh. A total of 3060 individuals’ data were collected from various sociodemographic backgrounds using a structured questionnaire followed by statistical analyses including Pearson’s chi-square, Spearman’s correlation, and logistic regression analysis, which were performed to assess the correlations and trends among variables using STATA and SPSS. The results revealed a significant heterogeneity in KPP across different sociodemographic groups. Importantly, the younger respondents (ages 21–30) indicated a high level of KPP, and respondents without formal education and belonging to older ages demonstrated markedly low levels of KPP. In the country’s divisional levels, respondents from Khulna and Rangpur revealed the highest and lowest KPP levels, respectively. Our study also revealed that students represented the major occupational demographic and displayed moderate levels of KPP. Notably, respondents highlighted the decline or absence of several wild animal species, including black bears, deer, vultures, wild cats, hanuman monkeys, and rhesus monkeys, from their local areas over the past decades. Public opinion on wildlife protection revealed 78.6% and 73.4% disapproving of hunting rejection and wildlife trading, respectively. Nonetheless, a lack of knowledge remained, as 16.89% of respondents were unaware of measures to alleviate human-wildlife conflict. The correlation analysis indicated a favorable association among KPP components, especially between knowledge and perception (correlation coefficient = 0.438), underscoring the essential influence of awareness on conservation actions. This study offers critical insights for developing interventions to enhance KPP among local communities and stakeholders, hence promoting sustainable wildlife conservation and biodiversity management in Bangladesh. Taken together, the findings provide baseline data for safeguarding biodiversity and fostering long-term wildlife sustainability in Bangladesh.

## Simple Summary

Human activities have profoundly impacted wildlife and biodiversity in Bangladesh, highlighting the necessity to enhance public knowledge, perceptions, and practices (KPP) for effective conservation efforts. This study analyzed data from 3060 individuals and revealed that younger respondents and those with formal education showed significantly higher KPP levels, whereas older and uneducated respondents revealed reduced KPP. In the region, Khulna demonstrated the highest KPP, whereas Rangpur reported the lowest. Respondents indicated significant decreases in wildlife species including black bears, deer, vultures, and monkeys, attributing these reductions to deforestation, urbanization, habitat encroachment, illegal hunting, and resource overexploitation. The current study emphasizes the pressing necessity for focused interventions to improve public KPP, which is crucial for promoting sustainable wildlife conservation and biodiversity management in Bangladesh.

## 1. Introduction

Human-wildlife relationships are currently experiencing significant changes on a global scale. Critical issues regarding the future of sustainable wildlife management and conservation are being prompted by these changes, which are being driven by evolving knowledge, perceptions, and practice (KPP) toward wildlife, as well as an increasing emphasis on compassionate conservation [1]. Knowledge equips individuals’ insights into socio-ecological systems and the significance of wildlife conservation, where perceptions influence attitudes and values toward conservation efforts, affecting support for legislation and programs [2,3]. On the other hand, the practices of individuals, such as minimizing habitat degradation, alleviating human-wildlife conflict, or engaging in conservation initiatives, are influenced by both knowledge and perception [4,5]. Therefore, optimal integration of these three components is crucial for establishing sustainable, community-oriented ways to safeguard biodiversity and facilitate the cohabitation of humans and wildlife within a balanced ecosystem. It is noteworthy that the KPP of individuals toward wildlife influences their willingness to co-exist with them, which can either facilitate or obstruct broader efforts to conserve biodiversity [6,7]. Previous studies have reported that habitat loss and forest degradation have considerably reduced biodiversity over the past five decades [8], and this decline continues in the presence of accelerating climate change [9]. Therefore, the reduction in plant biodiversity causes a reduction in animal biodiversity [10,11]. A study described that wildlife, human health, food security, and water availability are adversely affected by the loss of biodiversity, which leads to significant economic loss [12]. These disruptions further alter the whole biodiversity, thereby increasing the vulnerability of wildlife to invasive species and influencing wildlife species composition [12].

Bangladesh is located within the Indo-Burma biodiversity hotspot, which boasts of rich, diverse flora and fauna, underscoring its global ecological significance [13]. This geographical advantage, coupled with the country’s diverse ecosystems, makes it a significant area for wildlife conservation and biodiversity management efforts. However, beyond the conservation priorities, anthropogenic threats to biodiversity are particularly severe, which also often possess the fewest resources for conservation [14,15]. A study reported that Bangladesh faces significant environmental threats including pollution, deforestation, land appropriation, the disposal of industrial and domestic waste into water bodies, illegal hunting, and wildlife trafficking [16]. Bangladesh’s wildlife hunting and trafficking amounts to approximately US$ 10.5 million yearly [17,18]. Beyond this issue, Bangladesh’s wildlife conservation and biodiversity management is increasingly intricate and complicated by public matters. Previous studies reported that human activity plays a major role in impacting biodiversity and conservation disturbance [19,20,21]. These activities have degraded habitats, fragmented ecosystems, and strained natural resources, emphasizing the need for sustainable management to balance conservation and human needs. Therefore, understanding KPP is essential for sustainable wildlife conservation and biodiversity management, as it addresses the challenges of balancing conservation initiatives with sustainable use [22,23].

Previous studies have reported that many wildlife species, including tigers, black bears, Asian elephants, gaur, dhole, various species of civets, fishing cats, and sambar deer, are either almost extinct or significantly declining in various regions of Bangladesh [13,24,25]. It is challenging to pinpoint specific causes for this decline; rather, it appears to stem from a combination of factors, including habitat destruction, human-wildlife conflict, poaching, climate change, and insufficient KPP related to this [26,27]. To effectively address the challenges of wildlife conservation, we can interlock social data by integrating the public KPP into conservation strategies. Such a framework ensures that conservation efforts are inclusive, culturally sensitive, and grounded in scientific and social realities. Other studies reported that the KPP on wildlife and biodiversity among various individuals, including students, stakeholders, and local communities, frequently diverge significantly due to differences in age difference, educational backgrounds, access to knowledge, and cultural values, and to different degrees—it varies from country to country [28,29,30,31,32,33,34]. The disparities can affect the effectiveness of conservation methods, as the KPP of each group determines their contribution to conservation initiatives. Identifying these disparities is crucial for developing targeted interventions, encouraging inclusive involvement, and facilitating collaboration among varied groups to maintain the sustainability of wildlife conservation and biodiversity management. Studies reported that despite the efforts to implement wildlife laws and policies, the lack of integration of the public’s KPP remains a significant barrier to a successful conservation process in Bangladesh [34,35]. We hypothesize that a strategic evaluation of the public’s KPP related to wildlife conservation and biodiversity management can foster sustainable coexistence between humans and wildlife, enhance biodiversity conservation initiatives, and improve strategies to safeguard natural resources and ecosystems for long-term biodiversity sustainability in Bangladesh. However, no systematic research has been conducted on it yet. Thus, the current study aims to assess the general overview of the current levels of public KPP in Bangladesh regarding wildlife conservation and biodiversity management. To the best of our knowledge, this is the first study to find out the general overview of KPP regarding wildlife conservation and biodiversity management in Bangladesh.

## 2. Materials and Methods

### 2.1. Study Area

**Figure 1:**
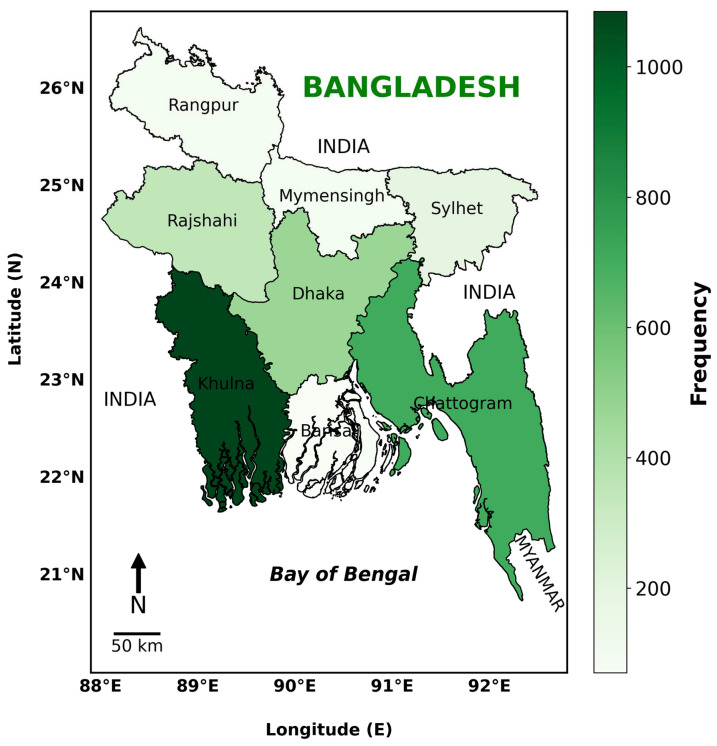
Study area in Bangladesh. The map shows a regional frequency distribution among the eight administrative divisions of Bangladesh: Rangpur, Rajshahi, Mymensingh, Sylhet, Dhaka, Khulna, Barishal, and Chattogram. A variety of green colors signifies frequency levels, with darker tones denoting greater events, as illustrated by the frequency scale bar on the right.

This study was conducted in the eight divisions of Bangladesh to get a broad understanding of the KPP of wildlife conservation and biodiversity management (Figure 1). The divisions encompassed several ecological zones, enabling a comprehensive analysis of perspectives and actions toward wildlife conservation across diverse environmental and socioeconomic contexts.

### 2.2. Informed Consent and Confidentiality

Before beginning the survey, census workers provided each participant with an overview of the study, clarified that participation was entirely optional, and obtained informed consent from the participants. The participants were assured that their responses would remain anonymous, which encouraged them to provide answers that were honest and detailed.

### 2.3. Data Collection

A cross-sectional survey was employed to gather data on KPP on wildlife conservation and biodiversity management. The study utilized a standardized questionnaire, developed in both English and Bengali, to ensure understanding among participants from various educational and socio-economic backgrounds. The data took over six months to collect to provide a comprehensive representation of the eight divisions and to accommodate participants’ schedules. The study examined a diverse population’s responses to investigate variations in KPP based on demographic factors. The respondents included local residents with various working classes, community leaders, students, government and NGO representatives, forest department officials, and indigenous communities living near wildlife areas. Stratified random sampling was utilized to ensure representation across several demographics, including age, education, occupation, and proximity to wildlife areas. This method enabled a fair distribution of survey participants from all backgrounds, providing a comprehensive general view of KPP in Bangladesh.

### 2.4. Survey and Questionnaires

The questionnaire included both closed and open-ended questions, enabling quantitative analysis and the gathering of qualitative insights (see the Supplementary Table S1 for detailed questionnaires). The survey employed both in-person interviews and self-administered questionnaires, depending on participant availability and interests. Participants in rural areas, remote locales, and those with limited literacy had in-person interviews. This strategy enabled the clarification of questions and ensured accurate data collection, especially for persons who may have struggled with written surveys. Interviews were conducted in Bengali to enhance understanding, and participants were encouraged to answer honestly. Prior to the survey, a group of trained enumerators received instruction on the study objectives, questionnaire structure, and ethical considerations. This ensured consistency and minimized interviewer bias, especially in rural or less literate demographics where face-to-face interviews were essential. The questionnaire was distributed in printed format in urban and metropolitan areas in all eight divisions, allowing informed individuals to complete it at their convenience. Field personnel were there to offer support for any required explanations, especially about technical vocabulary or specialized conservation concepts.

### 2.5. Data Analysis

The data was analyzed using both descriptive and inferential statistical techniques using STATA software ver. 17. Additionally, we utilized the Python packages ‘Numpy’ [36], ‘Pandas’ [37], ‘Seaborn’ [38], and ‘Matplotlib’ [39] to perform data analysis and visualization. Descriptive analysis such as frequencies, percentages, averages, and standard deviations (SD) were computed to assess sociodemographic data and responses to inquiries of KPP. Each participant’s responses were evaluated to generate an index of KPP levels. A scoring system was established by allocating points to each response, with elevated scores signifying KPP in wildlife conservation and biodiversity management. A Chi-square test was used to evaluate the relationships between sociodemographic variables and KPP regarding wildlife management and conservation. Logistic regression was also used to analyze the relationship between the tested variables to ascertain predictors of favorable KPP using STATA and SPSS. The study map was created using the Python packages ‘Cartopy’ (v0.22) and ‘Matplotlib’ [39], with shapefile data sourced from the Global Administrative Areas (GADM) database [40].

## 3. Results

### 3.1. Sociodemographic Characteristics of the Respondent

**Figure 2:**
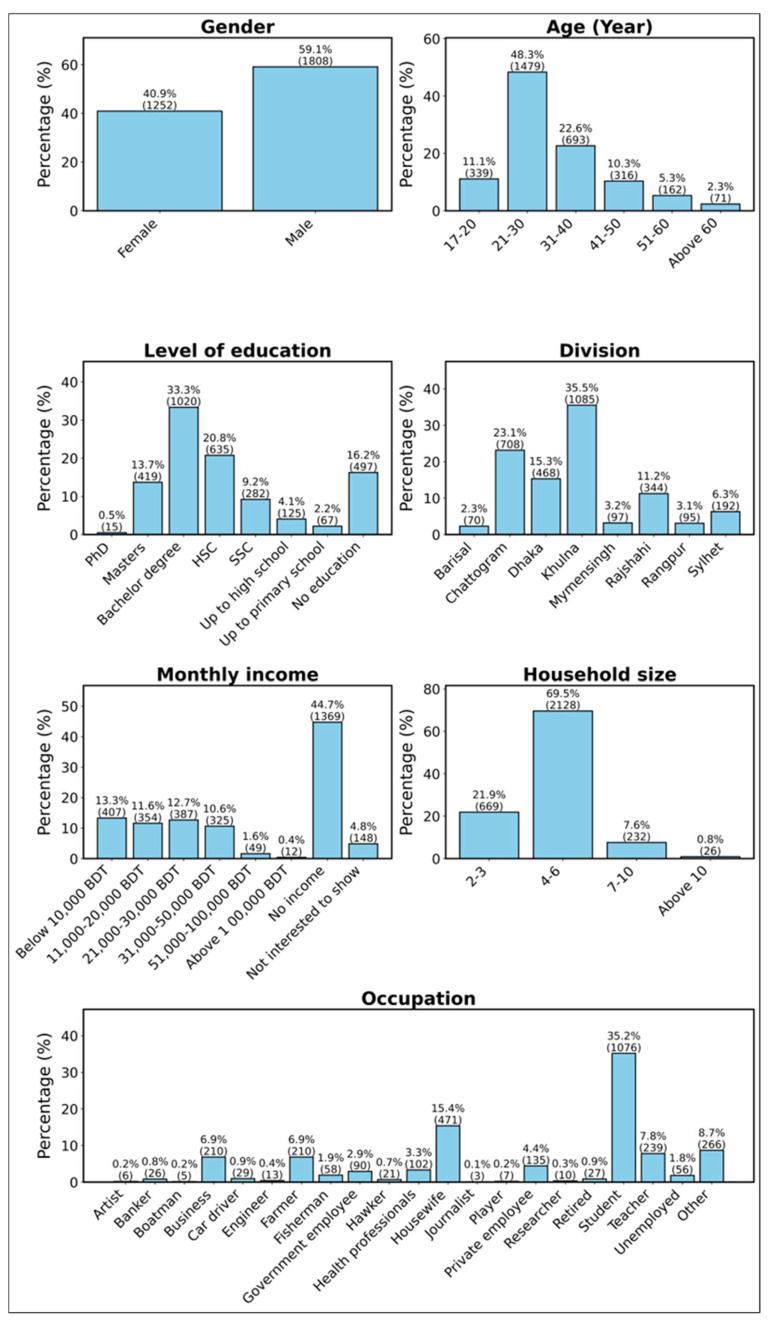
Socio-demographic characteristics of the respondents.

The demographic results included a diverse sample of 3060 respondents, covering a broad range of sociodemographic backgrounds to provide insights into the KPP related to wildlife management and conservation awareness across Bangladesh (Figure 2). In terms of gender, male participants constituted the majority at 59.1% (*n* = 1808), while female participants accounted for 40.9% (*n* = 1252). Regarding age distribution, the largest group was aged 21–30 years, representing 48.3% (*n* = 1479) of the respondents, followed by the 31–40 age group at 22.6% (*n* = 693). Participants aged 17–20 years made up 11.06% (*n* = 339), while those aged 41–50 accounted for 10.3% (*n* = 316). Smaller proportions were observed in the 51–60 age group at 5.3% (*n* = 162) and those above 60 years at 2.4% (*n* = 71). In terms of education level, the highest proportion of respondents had completed bachelor’s degrees (33.30%, *n* = 1020), followed by higher secondary school graduates (20.7%, *n* = 635) and master’s degree holders (13.7%, *n* = 419). Those with no institutional education constituted a significant portion at 16.2% (*n* = 497). Other levels included secondary school graduates at 9.2% (*n* = 282), up to high school at 4.1% (*n* = 125), up to primary school at 2.2% (*n* = 67), and PhD holders at 0.5% (*n* = 15). The divisional distribution of respondents showed that the highest and lowest representation were from Khulna and Barisal, accounting for 35.4% (*n* = 1085) and 2.3% (*n* = 70), respectively. The respondents from Chattogram were 23.1% (*n* = 708), Dhaka (15.3%, *n* = 469), Rajshahi (11.2%, *n* = 344), Sylhet (6.3%, *n* = 192), Mymensingh (3.2%, *n* = 97), and Rangpur (3.1%, *n* = 95). The monthly income distribution shows significant variation across the respondents. A significant percentage of individuals (44.7%, *n* = 1369) reported having no income, which is largely attributed to the substantial representation of students within the surveyed population. In contrast, 13.3% (*n* = 407) earn below 10,000 BDT, reflecting a lower-income group. Middle-income categories are represented by 11.6% (*n* = 354) earning between 11,000–20,000 BDT, 12.7% (*n* = 387) earning between 21,000–30,000 BDT, and 10.6% (*n* = 325) earning between 31,000–50,000 BDT. Additionally, 4.8% (*n* = 148) did not provide specific income data, adding to the incomplete data set. The survey results indicate that the majority of households consist of 4–6 members (69.5%, *n* = 2128), followed by smaller households with 2–3 members (21.9%, *n* = 669), while larger households have 7–10 members (7.6%, *n* = 232) and those with over 10 members (0.8%, *n* = 26) are relatively uncommon. In terms of occupation, students represent the largest group, comprising 35.2% (*n* = 1076), reflecting a significant academic demographic. The housewives account for 15.4% (*n* = 471), followed by teachers (7.8%, *n* = 239) and unemployed individuals (8.7%, *n* = 266). Other notable occupations include private employees (4.4%, *n* = 135) and researchers (0.9%, *n* = 27). Smaller proportions are observed among farmers (6.9%, *n* = 210), fishermen (6.9%, *n* = 210), and government employees (2.9%, *n* = 90). This diverse demographic distribution highlights a broad spectrum of perspectives across different divisions, age groups, and educational levels.

### 3.2. Public Knowledge About Biodiversity of Wild Animal Species

**Figure 3:**
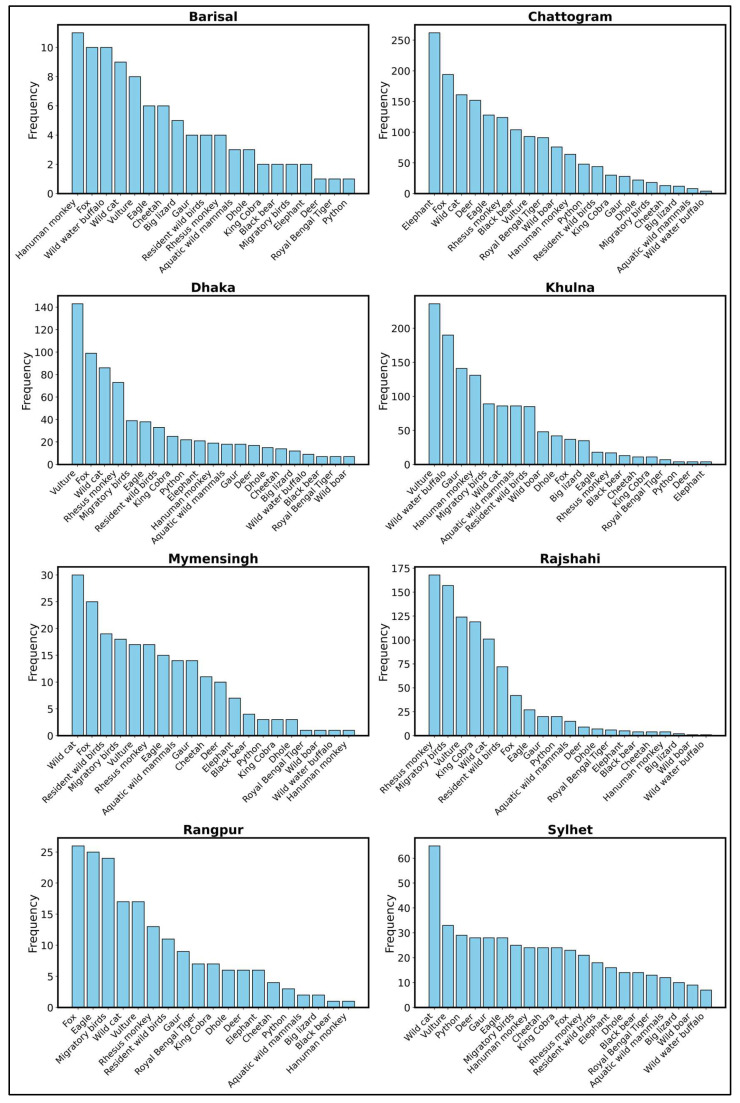
Respondent perceptions regarding the observed or declining wild animal species across eight divisions in Bangladesh: Barisal, Chattogram, Dhaka, Khulna, Mymensingh, Rajshahi, Rangpur, and Sylhet. Each graph highlights species that were once prevalent but are now rarely seen or have experienced significant declines over recent decades.

The result illustrates the frequency of observed or declining wild animal species across the eight divisions of Bangladesh, including Barisal, Chattogram, Dhaka, Khulna, Mymensingh, Rajshahi, Rangpur, and Sylhet, as reported by respondents (Figure 3). Vultures, foxes, and wild cats emerge as the most affected species, with their populations drastically declining across all divisions. In Barisal, in addition to hanuman monkeys, foxes, vultures, wild cats, and aquatic wild mammals are also rarely observed. Chattogram highlights the sharp decline of elephants, foxes, vultures, wild cats, gaurs, and wild water buffalo. Similarly, in Dhaka, vultures, wild cats, rhesus monkeys, and migratory birds are among the most critically affected, alongside resident birds, eagles, and king cobras. In Khulna, vultures, wild water buffalo, and hanuman monkeys have seen significant declines, along with gaur and migratory birds. Mymensingh also shows a similar trend, where vultures and wild cats are among the least observed, alongside foxes, migratory birds, and vultures. In Rajshahi, rhesus monkeys, migratory birds, and vultures lead the list of declining species, followed by king cobras and wild cats. In Rangpur, foxes, eagles, migratory birds, and vultures are rarely reported, along with wild cats and rhesus monkeys. Finally, in Sylhet, wild cats, vultures, pythons, and deer are noted as the most declining species.

### 3.3. Knowledge About Wildlife and Biodiversity

**Figure 4:**
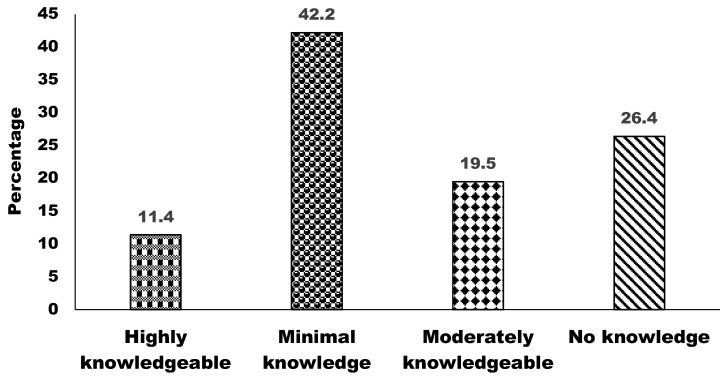
Respondents’ level of knowledge of wildlife and biodiversity.

The results on the level of knowledge about wildlife and biodiversity indicate varied levels of awareness among respondents (Figure 4). A significant proportion, 42.2%, reported having minimal knowledge, suggesting a basic or limited understanding of wildlife and biodiversity-related topics. Additionally, 26.4% of respondents indicated no knowledge, reflecting a lack of awareness in this area. In contrast, 19.5% of respondents were identified as being moderately knowledgeable, showing a fair level of understanding. The results also showed that 11.4% of respondents reported being highly knowledgeable, indicating an in-depth awareness and understanding. These findings highlight the need for targeted educational efforts to enhance knowledge, particularly among those with minimal or no awareness.

### 3.4. Changes in Wildlife Habitats in Bangladesh

**Figure 5:**
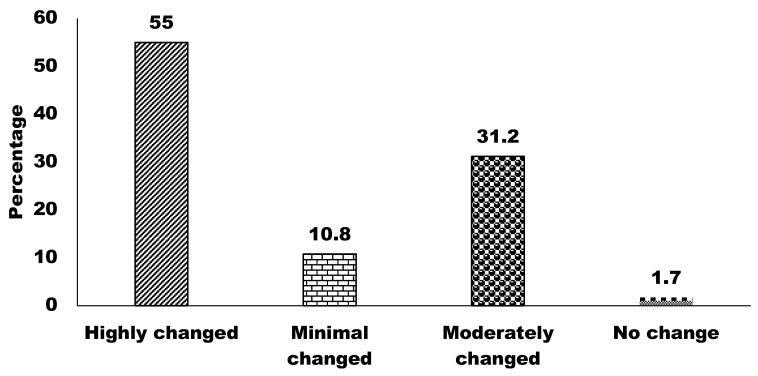
Level of changes in Bangladesh’s wildlife habitats over the last two decades.

The results from Figure 5 revealed that the majority of respondents, 55%, perceive wildlife habitats in Bangladesh to have undergone highly significant changes over the past two decades. The results also showed that 31.2%, 10.8%, and 1.7% of respondents reported moderate, minimal, and no changes, respectively, to wildlife habitats (Figure 5). These findings highlight a widespread acknowledgment of substantial habitat changes as indications of impacts from urbanization, deforestation, and other human-induced factors.

### 3.5. Perception of Causes of Destruction of Wildlife Habitat and Biodiversity in Bangladesh

**Figure 6:**
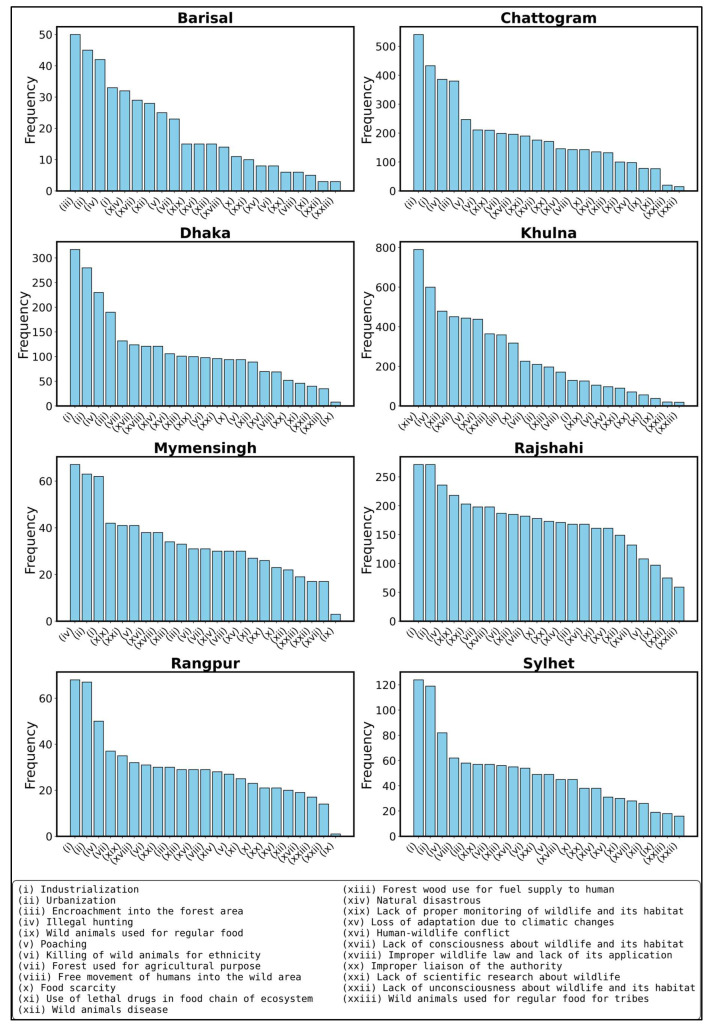
Respondents’ perception regarding the causes of destruction of wildlife and biodiversity in Bangladesh. The bar charts illustrate the frequency of responses for various causes across the eight divisions of Bangladesh: Barisal, Chattogram, Dhaka, Khulna, Mymensingh, Rajshahi, Rangpur, and Sylhet.

The results revealed the perceived causes of wildlife and biodiversity destruction across different divisions of Bangladesh, highlighting both common and region-specific challenges (Figure 6). The most commonly identified causes include industrialization, urbanization, and encroachment into forest areas, with these threats consistently observed across all divisions in Bangladesh. Briefly, in Barisal, additional concerns such as lack of consciousness about wildlife and improper monitoring of habitats exacerbate the destruction of biodiversity. In Chattogram, urbanization and forest encroachment are dominant threats, coupled with significant mentions of illegal hunting, poaching, and human-wildlife conflict, which have increasingly disrupted the ecosystem. Dhaka demonstrates a strong emphasis on industrialization, urbanization, and inadequate enforcement of wildlife laws, reflecting the pressures of rapid urban growth. Khulna is heavily impacted by natural disasters, human-wildlife conflict, and the extensive use of forest wood for fuel, which puts additional strain on already fragile habitats. In Mymensingh, illegal hunting, poaching, and food scarcity for wildlife emerge as critical challenges, indicating a lack of sustainable practices. Rajshahi exhibits the improper liaison among authorities and the lack of scientific research as major factors undermining conservation efforts. Similarly, in Rangpur and Sylhet, industrialization, human encroachment, and the lack of public awareness about wildlife pose significant threats to biodiversity. These findings underscore the pressing necessity for region-specific policies, focused educational activities, and enhanced policy enforcement to tackle the fundamental causes of wildlife habitat and biodiversity degradation.

### 3.6. Strategies to Avoid Human-Wildlife Conflict

Table 1 shows the strategies to mitigate human-wildlife conflict. The most common strategy, suggested by 27.71% of respondents, is keeping people out of wildlife areas. This was closely followed by increasing awareness of wildlife and its importance to biodiversity, which was also supported by 24.86% of respondents. In addition, 17.0% and 8.66% of respondents suggested an increase in protected areas for wild animals and local wildlife conservation efforts, respectively (Table 1). The result also included increasing coexistence between people and wildlife through necessary training (2.18%) and avoiding encroachment on wildlife habitats, nesting areas, and feeding grounds (0.13%). A notable 16.89% of respondents had no ideas about effective strategies, and 2.50% reported making no attempts to address the issue. These findings highlight the importance of awareness and habitat protection as key strategies to reduce human-wildlife conflict, while also emphasizing the need for educational initiatives to address knowledge gaps in effective conflict mitigation measures.

### 3.7. Perceptions of the Balance Between Humans and Wildlife

**Figure 7:**
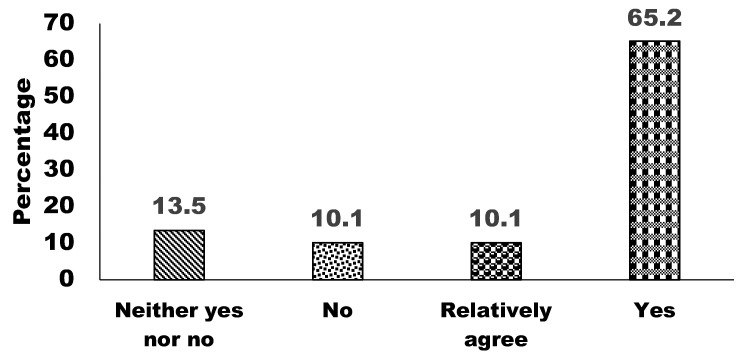
Respondents’ views on the relationship between humans and wildlife.

To assess the perceptions of the relationship between humans and wildlife, 65.2% of respondents agreed that humans and wildlife should coexist harmoniously, while 10.1% of respondents had partial support for this view (Figure 7). However, 10.1% and 13.5% of respondents remained disagreed and neutral (neither agreeing nor disagreeing), respectively, indicating a mixed opinion. These findings highlight a predominant consensus advocating for coexistence, while a notable minority remains uncertain or opposed.

### 3.8. Practice in Wildlife Hunting

**Figure 8:**
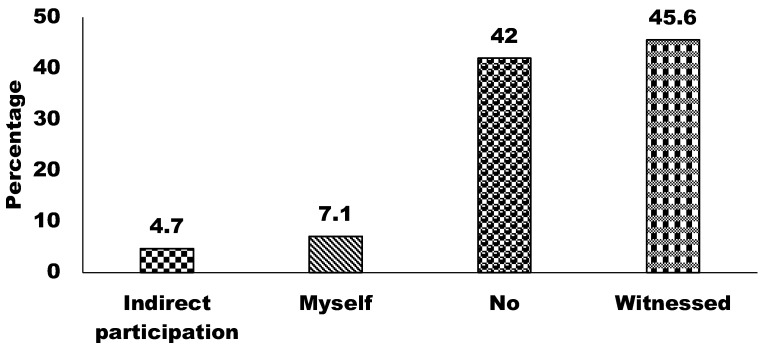
Participation in wildlife hunting.

Regarding practice in wildlife hunting, 45.6% of respondents reported having witnessed hunting, and 42% had no involvement or experience with hunting (Figure 8). In addition, 7.1% and 4.7% of respondents showed personal participation in hunting and indirect participation, either assisting or facilitating hunting activities, respectively. These findings suggest that direct participation in hunting is relatively low, while witnessed hunting is relatively high, indicating the visibility of this activity within the surveyed population.

### 3.9. Public Perception of Wildlife Hunting and Trade

To provide insights on public perceptions of wildlife hunting and trade, 78.60%, 11.60%, and 9.10% of respondents disagreed, agreed, and expressed neutrality, respectively, on whether wildlife hunting is a task that is right to do (Table 2). Similarly, 73.40%, 18.2%, and 7.7% of respondents opposed, supported, and remained neutral on wildlife trade (Table 2). Contrarily to the effects of illegal wildlife hunting, 60.8%, 14.7%, and 23.5% of respondents acknowledged, disagreed, and remained neutral on wildlife-related offenses. These findings indicated strong public disapproval of illegal wildlife hunting and wildlife trade, reflecting a conservation-oriented mindset among the majority, while highlighting some areas of neutral, mixed, or differing opinions that may require targeted educational efforts.

### 3.10. Association Between Sociodemographic Factors and Levels of KPP

Table 3 shows the significant associations between demographic factors and KPP of wildlife conservation and habitat management. The results show that younger age groups (21–30 years) and those that responded as having higher education levels (bachelor’s and master’s degrees) exhibited higher levels of knowledge, positive perception, and better practices (*p* < 0.001). Conversely, older age groups (over 51 years) and those with no formal education showed lower levels in all three areas. Moreover, divisional differences were also evident, with respondents from Khulna showing the highest levels of KPP, whereas respondents from Rajshahi and Sylhet exhibited lower levels. Students exhibited the highest levels of knowledge (19.86%) and positive perception (29.77%), followed by teachers and private employees (Table 3). In contrast, farmers, fishermen, and housewives showed lower levels, whereas boatmen and hawkers showed the lowest levels across KPP, indicating limited engagement with wildlife conservation. These findings underscore the influence of age, education, and geographic location on wildlife conservation awareness and engagement. 

### 3.11. Differences in KPP

The logistic regression analysis revealed significant associations between sociodemographic factors and levels of KPP of wildlife conservation and habitat management (Table 4). The result showed that younger age groups, particularly those aged 21–30 years, exhibited moderate odds ratio for knowledge (OR = 2.30, CI: 0.24–22.60), perception (OR = 2.41; CI: 0.40–14.41), and practice (OR = 2.16, CI: 0.37–12.73), with odds ratio decreasing in older age groups (61–70 years). The educational attainment positively influenced KPP, with PhD holders showing a higher odds ratio for perception (OR = 1.94, CI: 0.19–20.13), while respondents with no education had the lowest odds ratio across all categories: knowledge (OR = 0.03, CI: 0.02–0.05), perception (OR = 0.06, CI: 0.03–0.10), and practice (OR = 0.04, CI: 0.02–0.06). The divisional differences were significant, with respondents from Khulna showing higher odds ratio for knowledge (OR = 1.22, CI: 0.51–2.89) and practice (OR = 1.31, CI: 0.56–3.07), while those from Rangpur had significantly lower odds ratio, particularly for knowledge (OR = 0.18, CI: 0.07–0.47). These findings indicate the influence of age, education, and geographic location on KPP levels on wildlife conservation and habitat management. From the occupational point of view, the students showed moderate odds ratios for knowledge (OR = 0.46, CI: 0.01–17.13), perception (OR = 0.65, CI: 0.08–5.38), and practice (OR = 0.23, CI: 0.02–3.02). The housewives showed a moderate odds ratio for perception (OR = 3.01; CI: 0.31–34.04) but low odds ratios for knowledge and practice. Notably, the hawkers and fishermen consistently had low odds ratios across all categories, with hawkers showing the lowest odds ratio for practice (OR = 0.02, CI: 0.00–0.48). These findings highlight occupational disparities in KPP levels on wildlife conservation and habitat management.

### 3.12. Correlation Analysis Between KPP

**Figure 9:**
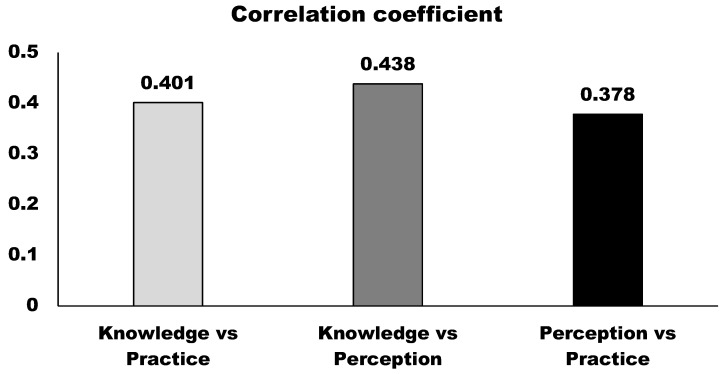
Spearman’s correlation analysis between KPP.

To determine correlations among KPP and variables related to wildlife conservation and habitat management, the strongest correlation was observed between knowledge and perception (correlation coefficient = 0.438), indicating that higher levels of knowledge are closely associated with more positive perceptions toward conservation (Figure 9). A moderate correlation was found between knowledge and practice (correlation coefficient = 0.401), suggesting that increased knowledge contributes to more active conservation practices. The weakest correlation was between perception and practice (correlation coefficient = 0.378), indicating that while positive perceptions influence conservation actions, the relationship is comparatively weaker. These results highlight the critical role of knowledge in shaping both attitudes and perceptions, emphasizing the importance of awareness and education programs to foster effective conservation behaviors.

## 4. Discussion

Bangladesh hosts a rich diversity of wildlife, including amphibians, birds, reptiles, and mammals, which hold significant ecological and cultural value [13]; however, this biodiversity faces severe threats from human-induced problems associated with a growing population [41,42]. Understanding the public’s KPP related to wildlife conservation and biodiversity management is essential to bridge existing gaps and promote sustainable coexistence. This study provides a foundation for addressing these critical needs to comprehend the assessment of the KPP on wildlife conservation and biodiversity management in Bangladesh. The current study revealed a notable decrease in numerous wildlife species throughout Bangladesh, as reported by respondents from various divisions. This includes vultures, wild cats, black bears, Hanuman monkeys, Asian elephants, gaurs, king cobras, foxes, aquatic mammals, and pythons, all of which have experienced significant population decreases. Previous studies reported many wild animal species such as Asian elephants, gaur, dhole, tigers, Indo-Chinese clouded leopards, fishing cats, sambar deer, and hoolock gibbons were found in Sylhet, Chattogram, Mymensingh, and Khulna regions of Bangladesh [24,26,43,44]. However, the public’s responses aligned positively with the International Union for Conservation of Nature (IUCN) data, identifying the low observance of several wild animal species from local areas. The IUCN (2015) [45] reported that several wild animal species in Bangladesh are either extinct or critically endangered. The report mentioned that extinct species include the Indian Javan rhinoceros and wild water buffalo, and critically endangered species include the royal Bengal tiger, Asian elephant, white-rumped vulture, and red-headed vulture. The findings indicate that the public’s KPP on biodiversity observation is largely reliable, closely corresponding with the wider conservation difficulties. This alignment indicates a commendable understanding among respondents concerning the status and decrease of wildlife species and biodiversity in their localities. It is well-demonstrated that the people are not only aware of the current biodiversity crisis but can also recognize endangered species, rendering their contributions significant for conservation strategies. This insight underscores the vital need for public KPP in formulating effective conservation measures. Our recent study identified public KPP regarding the several causes of the wildlife and biodiversity loss in Bangladesh, including industrialization, urbanization, forest encroachment, poaching, hunting, and the killing of wild animals, human-wildlife conflict, natural disasters, food scarcity, poor enforcement of wildlife laws, inadequate monitoring, lack of public awareness, and limited scientific research, which have hindered effective conservation efforts. Previous studies reported that several key factors driving wildlife and biodiversity loss in Bangladesh included habitat destruction due to deforestation, agricultural expansion, urbanization, overexploitation of resources through illegal logging, hunting, and fishing, which exacerbates the decline of species, as well as pollution from industrial, agricultural, and household waste [35,46,47]. Other studies reported that influential individuals and local communities are major contributors to the degradation of protected areas in Bangladesh by extracting resources such as timber and firewood for personal and commercial purposes [48,49]. The public’s KPP observed in our present study is consistent with these findings, reinforcing the alignment between KPP and the reported causes of wildlife and biodiversity loss in Bangladesh.

Our study revealed that 42.2% of respondents had minimal knowledge of wildlife and biodiversity, while 26.4% reported no knowledge, indicating a lack of awareness. In contrast, 19.5% demonstrated a moderate level of understanding. Knowledge is influenced by many factors beyond formal education, which is essential, as elevated educational attainment frequently correlates with enhanced comprehension and knowledge of wildlife and biodiversity [50]. Exposure to particular conservation education, whether from formal curriculum or extracurricular initiatives, augments knowledge by equipping individuals with the necessary tools and information to address environmental concerns. Chowdhury et al. (2014) [51] reported positive KPP among local communities regarding wildlife, particularly in the Rema-Kalenga Wildlife Sanctuary, Bangladesh. Another study reported on positive public practices to wildlife habitats, with 85% of respondents in Nepal favoring conservation areas [52]. In addition, 68% of respondents supported park management in Chitral Gol National Park, and 97.1% of respondents were willing to participate in wildlife protection in Pakistan [53]. However, the current study revealed a substantial portion of respondents lacked knowledge about wildlife and biodiversity, while nearly half of the respondents demonstrated minimal understanding. This suggested a critical gap in public KPP, indicating that a large segment of the population is either unaware or insufficiently informed about the importance of wildlife conservation and biodiversity management. These findings emphasize the pressing need for comprehensive educational programs and awareness campaigns to address these lacking and foster a more informed and engaged public. In our study, the majority of respondents were young people and females. Young respondents who have formal education demonstrated relatively higher levels of KPP regarding wildlife conservation and biodiversity compared to their counterparts, young respondents without education, and other age groups. Similarly, female respondents represented a significant proportion of the population, actively engaging in conservation discussions. While some portion of females showed strong understanding and positive attitudes, a considerable number of females showed limited knowledge, emphasizing the need for tailored programs to enhance their awareness and involvement. These findings underscore the importance of prioritizing young individuals and women in conservation outreach efforts to build a more informed and engaged community for biodiversity management. Age is a crucial factor, as younger generations may have higher exposure to contemporary conservation beliefs, whereas older individuals may depend more on traditional ecological knowledge [50,54]. Gender significantly influenced respondents’ opinions of nature and particular animals. Although men constituted the predominant group of respondents in our survey, women had more favorable opinions about animals. Chowdhury et al. (2014) [51] underscored that although Bangladeshi women possess restricted decision-making authority, they actively engage in forestry through resource extraction and maintenance, indicating the necessity to tackle gender inequality by promoting their involvement in co-management initiatives. This disparity in perception may be ascribed to women’s increased trepidation over animals, as indicated by another research [55,56,57]. This anxiety may arise from women’s infrequent contact with wildlife, in contrast to men, who typically engage with wildlife more directly, especially in contexts related to safeguarding families and cattle [55,58]. This gender disparity highlights the necessity of acknowledging varied experiences and engagements with animals in conservation awareness and educational programs.

The present study also reported human-wildlife conflicts and supported strategies to mitigate problems, including restricting human access to wildlife areas, enhancing awareness of wildlife conservation and biodiversity management, and educating the public on the importance of habitat preservation. However, a significant portion of respondents lacked knowledge of mitigation strategies, and some reported no active engagement in conflict resolution efforts, revealing substantial gaps in awareness and practice. Previous studies have highlighted that human-wildlife conflict and biodiversity management strategies encompass improving local communities’ attitudes and perceptions toward protected areas and their wildlife [59,60,61]. Effective conflict reduction necessitates collaborative endeavors across stakeholders, including policymakers, conservationists, and local communities, to foster sustainable cohabitation and guarantee long-term biodiversity preservation. The hunting and trade of wildlife are closely linked to the livelihoods of local communities, providing both food and money through associated activities. Hunting behaviors and views vary due to cultural, economic, and geographical influences [32]. In our current study, a significant percentage of respondents indicated having observed hunting activities, with some reporting direct or indirect participation. Furthermore, the majority of participants in our current survey indicated opposition to wildlife hunting and trade, whilst a lesser proportion endorsed these activities or maintained a neutral position. These data indicate the ongoing prevalence of hunting in local populations in Bangladesh, frequently motivated by socio-economic dependencies, traditional customs, and restricted choices. Previous study indicates the substantial impact of proximity to forested or protected areas on hunting behavior and perceptions of its legality, suggesting that residents residing closer to or within these areas tend to depend more on wildlife resources for their sustenance [62,63]. Combating these challenges necessitates a holistic strategy that incorporates sustainable livelihood initiatives, focused educational programs, and community involvement to diminish reliance on wildlife resources, promote beneficial conservation practices, and facilitate the coexistence of human and wildlife populations to guarantee biodiversity preservation. The present study encompasses an important group of respondents from the student demographic, indicating the essential impact that students exert on the future of wildlife conservation and biodiversity management in Bangladesh. Previous studies have indicated that students are uniquely equipped to contribute to conservation efforts owing to their access to educational resources, current knowledge, and opportunities for skill enhancement, which reflect their comprehension and endorsement of conservation, thereby establishing a foundation for future strategies [64,65,66,67]. Our findings indicated that students exhibit a fundamental comprehension of wildlife conservation, including the significance of wildlife, the protection of species and ecosystems, the intrinsic relationship between wildlife and humans, and the classification of conservation sites. Students had a favorable opinion of the necessity of protecting all species, regardless of their threat level or conservation status, indicating a robust basis for promoting proactive conservation actions. These findings are consistent with previous studies that emphasized the significance of tailored education to address the gaps in students’ roles in wildlife conservation and habitat management. Ultimately, the high number of students in this study not only demonstrates their potential as future stewards but also the significance of providing them with the education, information, resources, and drive required to spearhead Bangladesh’s next stage of wildlife conservation and biodiversity management.

This study provides significant insights into public KPP about wildlife conservation and biodiversity management in Bangladesh. Despite its strengths, this study possesses a few drawbacks. First, respondents were not preselected for interviews, possibly creating selection bias and constraining the range of responses. Second, difficulties in gathering data from local communities next to wildlife areas, due to social and logistical barriers such as distrust, cultural sensitivities, and limited access, hampered the incorporation of essential viewpoints. This is essential, as these groups are vital in influencing human-wildlife interactions and executing contextually appropriate conservation efforts. Third, the study’s cross-sectional design offers a snapshot of public KPP, omitting longitudinal trends and temporal variations. Fourth, the lack of previous research in Bangladesh hindered the comparison of findings with existing data, constraining the capacity to contextualize and elaborate on the topic. Fifth, although the study offers a comprehensive perspective, it fails to explore specific socio-economic or cultural factors that shape public perceptions, which could yield a more nuanced comprehension of conservation difficulties and potential. However, the results provide a robust basis for subsequent study and policy initiatives. They highlight the significance of specialized educational initiatives, community involvement, and localized conservation strategies to bridge knowledge gaps, address socio-economic challenges, and develop an informed and engaged public that promotes wildlife conservation and habitat management.

## 5. Conclusions

This preliminary investigation provides valuable insight into the general overview of the public’s KPP with sociodemographic factors regarding wildlife conservation and biodiversity management in Bangladesh. The study revealed significant disparities across age groups, education levels, and regions in Bangladesh. The study reported important findings that respondents had seen the noticeable decline or disappearance of several wild animal species, such as vultures, black bears, deer, wild cats, and several monkey species, from their local areas over the past two decades. Moreover, KPP on causes of wildlife destruction revealed urbanization, industrialization, encroachment into forest areas, and human-wildlife conflict had regional differences influencing the perceived causes and priorities for conservation efforts. Occupational differences further influenced KPP, with students and teachers showing greater awareness and involvement in conservation practices compared to other occupational groups. Additionally, younger age groups, particularly those aged 21–30 years and individuals with higher educational attainment demonstrated higher levels of KPP; in contrast, older age groups and respondents with no formal education exhibited significantly lower KPP levels, underscoring the need for targeted awareness and education initiatives in these populations. The present study underscores the necessity of tailored educational programs and policy interventions, particularly in wildlife-prone areas and local communities. The study also suggests the implementation of action plans at both local and national levels, fostering collaboration among governmental bodies, non-governmental organizations, and communities to enhance conservation awareness. Public understanding can be further improved through targeted education and awareness campaigns, fostering greater support for wildlife conservation and promoting sustainable practices to protect biodiversity in Bangladesh. This is the first study of KPP of wildlife conservation and biodiversity management in Bangladesh.

## Figures and Tables

**Table 1:** Strategies to avoid human-wildlife conflict.

Variable	Frequency	Percentage
Increasing protected areas for wild animals	521	17.02
Support local wildlife conservation efforts	265	8.66
Keeping people out of wildlife areas	702	27.71
Increasing the awareness about wildlife and its necessity in biodiversity	761	24.86
Avoid encroaching on wildlife habitats, nesting areas, and feeding grounds	4	0.13
Increasing coexistence between people and wildlife, giving necessary training	67	2.18
No attempt	77	2.50
No idea	517	16.89

**Table 2:** Public perception on wildlife hunting and trade.

	Yes	No	Either Yes or No
Variable	Freq (%)	Freq (%)	Freq (%)
Wildlife hunting is a task that is right to do	354 (11.60)	2408 (78.60)	278 (9.10)
Support wildlife trade	557 (18.20)	2247 (73.40)	237 (7.70)
Illegal wildlife hunting (wildlife-related offenses)	1863 (60.80)	450 (14.70)	721 (23.5)

**Table 3:** The variation in the respondents’ KPP of wildlife conservation and habitat management, based on their characteristics.

		Knowledge		Chi2	Perception		Chi2	Practice		Chi2
Variable	Category	Low Knowledge	High Knowledge	*p*-Value	Bad	Good	*p*-Value	Bad	Good	*p*-Value
Age	17–20	141 (4.60)	191 (6.24)	0.0001	93 (3.03)	242 (7.90)	0.0005	84 (2.74)	249 (8.13)	
	21–30	649 (21.20)	827 (27.02)		308 (10.06)	1469 (48.00)		338 (11.04)	1136 (37.09)	
	31–40	283 (9.24)	409 (13.36)		284 (9.28)	408 (13.33)		181 (5.91)	511 (16.69)	
	41–50	186 (6.07)	130 (4.24)		202 (6.60)	113 (3.69)		137 (4.47)	179 (5.84)	
	51–60	112 (3.66)	49 (1.60)		128 (4.18)	33 (1.07)		80 (2.61)	82 (2.67)	
	Above 60	47 (1.53)	12 (0.39)		49 (1.60)	9 (0.29)		27 (0.88)	33 (1.07)	
Education	PhD	5 (0.166)	8 (0.26)		1 (0.03)	14 (0.45)		15 (0.49)	15 (0.49)	
	Masters	132 (4.31)	287 (9.37)		48 (1.56)	371 (12.12)		404 (13.20)	417 (13.62)	
	Bachelor	348 (11.36)	479 (15.64)		119 (3.88)	705 (23.03)		717 (23.42)	826 (26.98)	
	HSC	248 (8.10)	386 (12.61)		145 (4.73)	487 (15.91)		475 (15.52)	633 (20.68)	
	SSC	106 (3.46)	172 (5.62)		141 (4.60)	139 (4.54)		176 (5.75)	279 (9.11)	
	Up to high school	78 (2.54)	46 (1.50)		93 (3.03)	32 (1.04)		63 (2.05)	125 (4.08)	
	Up to primary school	45 (1.47)	22 (0.71)		49 (1.60)	18 (0.58)		26 (0.84)	67 (2.18)	
	No education	432 (14.11)	64 (2.09)		435 (14.21)	54 (1.76)		156 (5.09)	497 (16.24)	
Division	Barisal	11 (0.35)	59 (1.92)		13 (0.42)	57 (1.86)		4 (0.13)	66 (2.15)	
	Chattogram	371 (12.11)	334 (10.90)		238 (7.80)	468 (15.28)		300 (9.80)	404 (13.20)	
	Dhaka	178 (5.81)	288 (9.41)		119 (3.88)	348 (11.37)		61 (1.99)	404 (13.20)	
	Khulna	398 (13.00)	686 (22.41)		493 (16.11)	590 (19.28)		364 (11.89)	721 (23.56)	
	Mymensingh	55 (1.79)	42 (1.37)		16 (0.52)	81 (2.64)		10 (0.32)	87 (2.84)	
	Rajshahi	238 (7.77)	103 (3.36)		122 (3.98)	221 (7.22)		45 (1.47)	298 (9.73)	
	Rangpur	50 (1.63)	44 (1.43)		23 (0.75)	72 (2.35)		4 (0.13)	91 (2.97)	
	Sylhet	128 (4.18)	64 (2.09)		49 (1.60)	132 (4.32)		65 (2.12)	126 (4.11)	
Occupation	Banker	7 (0.22)	19 (0.62)		3 (0.09)	4 (0.13)		4 (0.13)	22 (0.71)	
	Boatman	4 (0.13)	1 (0.03)		5 (0.16)	1 (0.03)		3 (0.09)	2 (0.06)	
	Business	61 (1.99)	148 (4.83)		87 (2.84)	123 (4.01)		25 (0.81)	185 (6.04)	
	Car driver	16 (0.52)	13 (0.42)		22 (0.71)	7 (0.22)		10 (0.32)	19 (0.62)	
	Engineer	1 (0.03)	12 (0.39)		1 (0.03)	12 (0.39)		1 (0.03)	12 (0.39)	
	Farmer	169 (5.52)	41 (1.33)		170 (5.55)	36 (1.17)		106 (3.46)	104 (3.39)	
	Fisherman	45 (1.47)	13 (0.42)		47 (1.53)	11 (0.35)		16 (0.52)	42 (1.37)	
	Govt employe	35 (1.14)	55 (1.79)		15 (0.49)	75 (2.45)		7 (0.22)	83 (2.87)	
	Hawker	19 (0.62)	1 (0.03)		19 (0.62)	2 (0.06)		13 (0.42)	8 (0.26)	
	Health professionals	31 (1.01)	71 (2.32)		4 (0.13)	98 (3.20)		2 (0.06)	100 (3.26)	
	Housewife	275 (8.98)	196 (6.40)		339 (11.07)	132 (4.31)		281 (9.18)	120 (3.92)	
	Journalist	1 (0.03)	2 (0.06)		1 (0.03)	2 (0.06)		1 (0.03)	2 (0.06)	
	Others	148 (4.83)	119 (3.88)		115 (3.75)	149 (4.86)		148 (4.83)	117 (3.82)	
	Player	1 (0.03)	6 (0.19)		2 (0.06)	5 (0.16)		3 (0.09)	4 (0.13)	
	Private employee	38 (1.24)	97 (3.16)		29 (0.94)	106 (3.46)		11 (0.35)	123 (4.01)	
	Researcher	3 (0.09)	7 (0.22)		1 (0.03)	9 (0.29)		1 (0.03)	9 (0.29)	
	Retired	16 (0.52)	11 (0.35)		15 (0.49)	10 (0.32)		7 (0.22)	20 (0.65)	
	Student	462 (15.09)	608 (19.86)		157 (5.13)	911 (29.77)		178 (5.81)	891 (29.11)	
	Teacher	64 (2.09)	174 (5.68)		22 (0.71)	217 (7.09)		7 (0.22)	232 (7.58)	
	Unemployed	35 (1.14)	21 (0.68)		19 (0.62)	35 (1.14)		28 (0.91)	27 (0.88)	

**Table 4:** Logistic regression analysis of the factors associated with respondents’ KPP on wildlife conservation and habitat management.

		Knowledge			Perception			Practice		
Variable	Category	Odds Ratio	95% CL		Odds Ratio	CI		Odds Ratio	CI	
			Lower	Upper		Lower	Upper		Lower	Upper
Age (years)	17–20	2.28	0.23	22.87	1.24	0.20	7.71	2.03	0.34	12.22
	21–30	2.30	0.24	22.60	2.41	0.40	14.41	2.16	0.37	12.73
	31–40	2.68	0.28	26.12	1.75	0.29	10.44	2.63	0.45	15.49
	41–50	1.95	0.20	19.07	1.34	0.22	8.05	1.79	0.30	10.61
	51–60	1.89	0.19	18.66	0.77	0.12	4.79	1.84	0.31	11.08
	Above 60	0.73	0.07	7.63	0.33	0.05	2.44	1.18	0.19	7.53
Educational qualification	PhD	1.85	0.18	18.68	1.94	0.19	20.13	1.76	0.20	15.18
	Masters	0.74	0.40	1.34	1.21	0.72	2.01	0.89	0.50	1.60
	Bachelor	0.94	0.48	1.85	1.09	0.61	1.99	1.05	0.54	2.01
	HSC	0.45	0.25	0.80	0.52	0.33	0.81	0.51	0.29	0.89
	SSC	0.38	0.21	0.70	0.23	0.14	0.37	0.43	0.24	0.78
	Up to high school	0.10	0.05	0.20	0.14	0.08	0.26	0.12	0.06	0.23
	Up to primary school	0.09	0.04	0.20	0.14	0.06	0.29	0.11	0.05	0.24
	No education	0.03	0.02	0.05	0.06	0.03	0.10	0.04	0.02	0.06
Division	Barisal	0.18	0.08	0.43	0.61	0.28	1.30	0.21	0.09	0.50
	Chattogram	0.42	0.18	1.01	0.57	0.02	15.23	0.30	0.02	4.81
	Dhaka	0.42	0.18	1.01	1.05	0.49	2.26	0.43	0.18	1.00
	Khulna	1.22	0.51	2.89	0.83	0.39	1.75	1.31	0.56	3.07
	Mymensingh	0.20	0.07	0.52	1.29	0.48	3.45	0.25	0.10	0.64
	Rajshahi	0.13	0.05	0.30	1.06	0.47	2.39	0.14	0.06	0.34
	Rangpur	0.18	0.07	0.47	0.49	0.20	1.21	0.22	0.09	0.57
	Sylhet	0.35	0.13	0.91	1.98	0.81	4.83	0.62	0.25	1.56
Occupation	Banker	0.24	0.01	6.45	0.89	0.09	9.19	0.52	0.03	10.36
	Businessman	0.17	0.01	3.14	0.48	0.07	3.58	0.22	0.02	3.02
	Car driver	0.12	0.01	2.58	0.44	0.05	3.87	0.15	0.01	2.29
	Fisherman	0.09	0.00	1.71	0.41	0.05	3.12	0.23	0.02	3.28
	Government employee	0.09	0.00	1.75	0.46	0.05	3.81	0.26	0.02	3.63
	Hawker	0.16	0.01	3.17	0.84	0.11	6.52	0.02	0.00	0.48
	Health professionals	0.02	0.00	0.56	0.17	0.01	2.44	0.68	0.05	9.75
	Housewife	0.62	0.02	12.30	3.01	0.31	34.04	0.17	0.00	4.68
	Private employee	0.15	0.01	2.72	0.53	0.07	3.89	0.41	0.03	5.88
	Researcher	0.24	0.01	9.61	0.94	0.07	12.56	0.54	0.02	15.17
	Retired	0.27	0.01	5.23	0.84	0.11	6.33	0.17	0.01	2.87
	Student	0.46	0.01	17.13	0.65	0.08	5.38	0.23	0.02	3.02
	Teacher	0.14	0.01	3.25	0.77	0.07	8.06	0.19	0.01	2.67
	Unemployed	0.16	0.01	3.06	0.98	0.13	7.26	0.20	0.01	2.90
	Others	0.09	0.00	1.64	0.12	0.02	0.93	0.26	0.02	3.52

## Data Availability

The data presented in this study are available within the article.

## Supplementary Materials

The following supporting information can be downloaded at: https://www.mdpi.com/article/10.3390/ani15030296/s1, Table S1: Questionnaire for the respondents.
