# Supplementary material for: Knowledge, Perception, and Practices of Wildlife Conservation and Biodiversity Management in Bangladesh
Source: Animals (Basel). 2025 Jan 21;15(3):296. doi: 10.3390/ani15030296 (PMC11816245; doi:10.3390/ani15030296)
Supplement: Supplementary file 1 [file animals-15-00296-s001.zip › animals-3430115-supplementary.pdf]

Table S1. Questionnaire for the respondents

1. Name of the respondent:
2. Sex: Male Female
3. Marital status:
   i. Unmarried
   ii. Married
4. Age:
   i. 17-20
   ii. 21-30
   iii. 31-40
   iv. 41-50
   v. 51-60
   vi. above 60
5. Educational qualification:
   i. PhD
   ii. Masters
   iii. Bachelor
   iv. Higher secondary certificate (HSC)
   v. Secondary school certificate (SSC)
   vi. Up to high school
   vii. Up to primary school
   viii. No institutional education
6. Occupation:
   i. Banker
   ii. Boatman
   iii. Business
   iv. Car driver
   v. Engineer
   vi. Farmer
   vii. Fisherman
   viii. Government employee
   ix. Hawker
   x. Health professional
   xi. Housewife
   xii. Journalist
   xiii. Player

xiv. Private employee

xv. Researcher

xvi. Retired

xvii. Student

xviii. Teacher

xix. Unemployed

xx. Others (if not mentioned above)

7. Monthly income (BDT):

i. Below 10,000

ii. 11,000-20,000

iii. 21,000-30,000

iv. 31,000-40,000

v. 41,000-50,000

vi. 50,000-100,000

vii. Above 100,000

viii. No income

ix. Not interested to show

8. Your division:

i. Barisal

ii. Chattogram

iii. Dhaka

iv. Khulna

v. Mymensingh

vi. Rajshahi

vii. Rangpur

viii. Sylhet

9. Number of members in your family:

i. 2-3

ii. 4-6

iii. 7-10

iv. Above 10

10. Does anyone in your family employed anywhere in related with wildlife:

i. Yes

ii. No

11. Is anyone in your household financially reliant on resources derived from wildlife or its habitat:

i. Yes

ii. Partially
iii. No

12. Do you have any knowledge about wildlife and biodiversity:
    i. Yes
    ii. No
    iii. Neither yes nor no
13. How would you rate your knowledge regarding the wildlife and biodiversity of Bangladesh:
    i. Highly knowledgeable
    ii. Moderately Knowledgeable
    iii. Minimal Knowledge
    iv. No Knowledge
14. Do you believe humans and wildlife should coexist harmoniously in nature?:
    i. Yes
    ii. No
    iii. Neither yes nor no
    iv. Relatively agree
15. Have you ever been to a zoo, national park, animal sanctuary, protected area, or safari park in Bangladesh:
    i. Yes
    ii. No
16. Do you have any wildlife zone or forest in your area:
    i. Yes
    ii. No
    iii. I don’t know
17. Have you noticed any changes in Bangladesh's wildlife habitat over the last 2 decades:
    i. Highly changed
    ii. Modernly changed
    iii. Minimal change
    iv. No change
18. Which wild animal species have declined or are rarely seen in your area:
    i. Hanuman monkey
    ii. Fox
    iii. Black bear
    iv. Cheetah
    v. Eagle
    vi. Wild cat

vii. Vulture
viii. Rhesus monkey
ix. Resident wild birds
x. Migratory birds
xi. Aquatic wild mammals
xii. King cobra
xiii. Python
xiv. Elephant
xv. Dhole
xvi. Deer
xvii. Civets
xviii. Gaur
xix. Royal Bengal tiger
xx. Wild Water Buffalo
xxi. Big lizards

19. What are your thoughts on the causes of the destruction of wildlife and biodiversity:
    i. Industrialization
    ii. ii. Urbanization
    iii. Encroachment into the forest area
    iv. Illegal hunting
    v. Wild animals used for regular food
    vi. Poaching
    vii. Killing of wild animals for ethnicity
    viii. Forest used for agricultural purposes
    ix. Free movement of humans into the wild area
    x. Food scarcity
    xi. Use of lethal drugs in the food chain of the ecosystem
    xii. Wild animal diseases
    xiii. Forest wood used for fuel supply to humans
    xiv. Natural disasters
    xv. Lack of proper monitoring of wildlife and its habitat
    xvi. Loss of adaptation due to climatic changes
    xvii. Human-wildlife conflict
    xviii. Lack of consciousness about wildlife and its habitat
    xix. Improper wildlife law and lack of its application
    xx. Improper liaison of the authority

xxi. Lack of scientific research about wildlife

xxii. Lack of unconsciousness about wildlife and its habitat

xxiii. Wild animals used for regular food for tribes

20. How worried are you about the various environmental threats of wildlife to Bangladesh:
    i. Highly concern
    ii. Moderately concern
    iii. Minimal concern
    iv. No concern
21. Is there any need for wildlife conservation to protect the overall ecological balance of the country:
    i. Highly important
    ii. Moderately important
    iii. Neutral
    iv. No need

22. Which of the following strategies would you like to take to avoid human-wildlife conflict:
    i. Increasing protected areas for wild animals
    ii. Supporting local wildlife conservation efforts
    iii. Keeping people out of wildlife areas
    iv. Increasing awareness about wildlife and its necessity in biodiversity
    v. Avoiding encroachment on wildlife habitats, nesting areas, and feeding grounds
    vi. Increasing coexistence between people and wildlife by providing necessary training
    vii. No attempt
    viii. I don't have any idea

23. Have you ever hunted wildlife or witnessed someone hunting wildlife:
    i. Myself
    ii. Witnessed
    iii. Indirect participation
    iv. No
24. Do you think hunting wildlife is a right task:
    i. Yes
    ii. Neither yes nor no
    iii. No
25. In several areas of Bangladesh, wild animals are frequently spotted being traded, do you support the trade of wildlife:

i. Yes
ii. Neither yes nor no
iii. No

26. Do you agree that wildlife habitat and biodiversity conservation could contribute for the sustainable development of Bangladesh:
    i. Highly agree
    ii. Moderately agree
    iii. Minimal agree
    iv. Highly disagree
27. How would hunting affects the ecological balance of wildlife:
    i. Highly harmful
    ii. Moderately harmful
    iii. Minimal harmful
    iv. Not harmful
28. Do you think that illegal wildlife hunting can cause other wildlife-related offense:
    i. Yes
    ii. No
    iii. Either yes or no
